# Innovations and Challenges in Reducing Maternal Mortality in Tamil Nadu, India

**DOI:** 10.3329/jhpn.v27i2.3364

**Published:** 2009-04

**Authors:** P. Padmanaban, Parvathy Sankara Raman, Dileep V. Mavalankar

**Affiliations:** ^1^ Public Health Administration, National Health Systems Resource Centre, National Rural Health Mission, Ministry of Health and Family Welfare, New Delhi, India; ^2^ Centre for Management of Health Services, National Health Systems Resource Centre, National Rural Health Mission, Ministry of Health and Family Welfare, New Delhi, India; ^3^ Public Systems Group, Indian Institute of Management, Vastrapur, Ahmedabad 380 015, India

**Keywords:** Maternal health, Maternal mortality, Obstetric care, Verbal autopsy, India

## Abstract

Although India has made slow progress in reducing maternal mortality, progress in Tamil Nadu has been rapid. This case study documents how Tamil Nadu has taken initiatives to improve maternal health services leading to reduction in maternal morality from 380 in 1993 to 90 in 2007. Various initiatives include establishment of maternal death registration and audit, establishment and certification of comprehensive emergency obstetric and newborn-care centres, 24-hour x 7-day delivery services through posting of three staff nurses at the primary health centre level, and attracting medical officers to rural areas through incentives in terms of reserved seats in postgraduate studies and others. This is supported by the better management capacity at the state and district levels through dedicated public-health officers. Despite substantial progress, there is some scope for further improvement of quality of infrastructure and services. The paper draws out lessons for other states and countries in the region.

## INTRODUCTION

Maternal and neonatal health has been priorities for the Government of Tamil Nadu for over a decade. This case study highlights the various initiatives and innovations carried out by the state for improving maternal health that resulted in a trend of reduction in the maternal mortality ratio (MMR) from 380 in 1993 to 90 in 2007 (1).

Tamil Nadu, the southern-most state of India, comprises 31 administrative districts, 73 revenue divisions, 10 municipal corporations, 385 blocks, and 17,244 villages (2). The purpose of this paper was to understand how Tamil Nadu has been able to reduce the MMR so rapidly.

## MATERIALS AND METHODS

This case study was developed based on the personal observations of one (PP) of the authors and a review of available literature and secondary data for Tamil Nadu. Secondary data were drawn from various national surveys and service statistics compiled by the State Government, including the three National Family Health Surveys (1992-1993, 1998-1999, and 2005-2006), family welfare statistics in India (2006), and other facility surveys conducted in India [District Level Household Survey (DLHS) 2002-2004]. Various documents on policy and programme and relevant literature were analyzed. Published and unpublished reports of government and non-governmental agencies were reviewed to gain insights into the maternal health situation in Tamil Nadu. The case study also uses personal observations by authors (DVM and PSR) made during their visits to various facilities in the state in July 2006. Discrepancies among various secondary sources of data due to different definitions and data-collection methods limit the study.

## RESULTS

### Socioeconomic and demographic profile

Tamil Nadu is the sixth most populous state of India with a population of 62.41 million (2001), contributing approximately 6% to the total population of India, and the eleventh most densely-populated state. In 2001, the density of its population was 478 persons per sq km, having increased from 429 in 1991; it is significantly more densely-populated than the Indian average of 324 persons per sq km. The population of Tamil Nadu grew by 11.19% between 1991 and 2001, the second lowest rate (after Kerala) for that period among populous states of India (states whose population exceeded 20 million in 2001). Its decadal rate of population growth has declined in every decade since 1971, one of only three populous states, along with Kerala and Orissa, to show this trend (3). The economy of the state is largely agriculture-based, although it is industrially well-developed. Table 1 shows the comparative demographic indicators for Tamil Nadu, India, and other comparable states. The percentage of those living below the poverty-line was 35% in 1993-2004 compared to the national figure of 36%. Between 1993-1994 and 1999-2000, poverty declined in Tamil Nadu to 21% against 26% nationally (4).

**Table 1.** Comparision of sociodemographic and health indicators for India, Tamil Nadu, Gujarat, Rajasthan, and Andhra Pradesh (5,6)

Indicator	All-India	Tamil Nadu	Gujarat	Rajasthan	Andhra Pradesh	Kerala
Population (million) (2001) (page A-9)	1,028	62.4	50.67	56.50	76.2	31.84
Sex ratio (2001) (page A-6)	933	987	920	921	978	1058
Decennial growth rate (%) (1991-2001) (page A-9)	21.54	11.19	22.66	28.41	14.59	9.43
Average annual exponential growth rate (%) (1991-2001)	1.93	1.06	2.03	2.49	1.30	0.9
Urban population as % of total population (2001) (page A-9)	27.8	44.4	37.36	23.9	27.30	25.96
Life expectancy for women (years) (2001-2006)∗	65.4	69.8	64.1	62.8	65.0	75.0
Effective age (years) at marriage (2005)	20.2	21.8	20.3	19.9	18.7	22.9
Literacy rate: total (2001)	65.3	73.4	69.1	60.4	60.4	90.92
Male	75.3	82.4	79.9	75.7	70.3	94.20
Female	54.1	64.4	57.8	43.8	53.7	87.87
Crude birth rate (2005) (page A-26 and 27)	23.8	16.5	23.7	28.6	19.1	15.0
Crude death rate (2005) (page A-28 and 29)	7.6	7.4	7.1	7.0	7.3	6.4
Infant mortality rate (2005) (page A-36)	58	37	54	68	57	14
Neonatal mortality rate (2005) (page A-37)	37	26	36	43	35	9
Total fertility rate (2005) (page A-41)	2.9	1.7	2.8	3.7	2.0	1.7
Maternal mortality ratio (2003) SRS estimates (page A:65)	301	134	172	445	195	110
% of births of order 3+ (DLHS 2004) (page A-79)	42.0	21.6	38.1	47.4	22.5	15.5
At least 3 ANC (NFHS 3) (page A:74)	50.7	96.5	64.9	41.2	86.0	93.9
Birth assisted by doctor/ nurse/LHV/ANM/other health personnel (NFHS 3) (page A:75)	48.2	93.2	64.7	43.2	74.2	99.7
Institutional births (%) (NFHS 3) (page A:75)	40.7	90.4	64.7	43.2	68.6	99.5
Pregnant women who are anaemic (NFHS 3) (page no: A77)	57.8	53.3	60.8	56.4	61.2	33.1
Full immunization (NFHS 3) (page A-75)	43.5	80.8	45.2	26.5	46.0	75.3

ANC=Antenatal care; ANM=Auxiliary Nurse Midwife; DLHS=District Level Household Survey; LHV=Lady Health Visitor; NFHS=National Family Health Survey; SRS=Sample Registration System

∗Source: State-wise life expectancy at birth by sex in India. (http://www.indiastat.com/india/ShowDataSec.asp?secid=7698&ptid=17797, accessed on 5 April 2009)

Table 1 shows that Tamil Nadu performs better in terms of demographic and socioeconomic indicators than India nationally and most other states of the country. The sex ratio of Tamil Nadu is high compared to the other states and also for the whole of India next only to Kerala. Approximately 44% of the population of Tamil Nadu lives in urban areas, much higher than the national average and also high compared to Gujarat, one of the more developed states in India. This rapid urbanization is a cause of concern for public-health specialists because of the increase in urban slums. The total fertility rate reduced from 2.1 in 2000 to 1.7 in 2005, possibly due to strong political commitment of governments to family planning, progressive socioeconomic movements of the past, sustained information programmes, increasing educational levels, rising aspiration, and the increase in the standard of life.

The institutional delivery rate of 90.7% is very high compared to the national average of 40.7% and is even higher than in Andhra Pradesh (68.6%), a neighbouring state. In Tamil Nadu, skilled birth attendants (SBAs) assist most (93.2%) deliveries compared to 68.6% in Andhra Pradesh. The infant and maternal mortality rates of Tamil Nadu are low compared to the national average and other states. The table shows that the state ranks the second highest in the health indicators, next only to the state of Kerala.

### Status of women

Although the Indian constitution grants women equal rights as men, strong patriarchal traditions still persist, with lives of women shaped by customs that are centuries old. In most Indian families, a daughter is viewed as a liability and conditioned to believe that she is inferior and subordinate to a son. Tamil Nadu is no exception to these traditions. Although sex-selective abortions are legally banned, it is still practised in some districts, such as Salem, Namakal, Madurai, and Dharmapuri. The sex ratio in Dharmapuri district is 952 (7), which is much higher than that in Gujarat or the national average. Female literacy is higher compared to the national rate. Although the mean age at marriage is also high compared to the national average (Table 1), it does not indicate that women enjoy better status in society. The decision-making power lies with the male counterpart for such matters as the size of the family and sex-selective abortions. Female child workers accounted for about 55% of the total child workers in 1987-1988 and 1993-1994. During 1999-2000, however, male child workers (56.3%) outnumbered female child workers (43.7%) (4). Table 2 shows the prevalence of anaemia among women of reproductive age for the last two National Family Health Surveys (NFHSs) (1998-1999 and 2005-2006); it still has not improved.

**Table 2.** Prevalence of anaemia among women of reproductive age, Tamil Nadu (6,8)

Survey	Mild anaemia	Moderate anaemia	Severe anaemia	Any kind of anaemia
NFHS 2 (1998-1999)	36.7	15.9	3.9	56.5
NFHS 3 (2005-2006)	37.4	13.6	2.2	53.2

NFHS=National Family Health Survey

## Findings

### Maternal health in Tamil Nadu

The state has become one of the top performers in the country in terms of maternal health with its MMR now at 90 (2007) (1) compared to other states (Table 3).

**Table 3.** Maternal mortality ratio in Tamil Nadu, Andhra Pradesh, Rajasthan, and Gujarat, 1997-2003 (9)

State	1997-1998	1999-2001	2001-2003
Andhra Pradesh	197	220	195
Gujarat	46	202	172
Rajasthan	508	501	445
Tamil Nadu	131	167	134
All-India	398	327	301

Reasons behind the decline in maternal mortality in Tamil Nadu are assumed to be the increase in institutional deliveries, deliveries assisted by a skilled birth attendant (SBA) and use of emergency obstetric care (EmOC) when required.

#### Safe deliveries

**Fig. 1.**
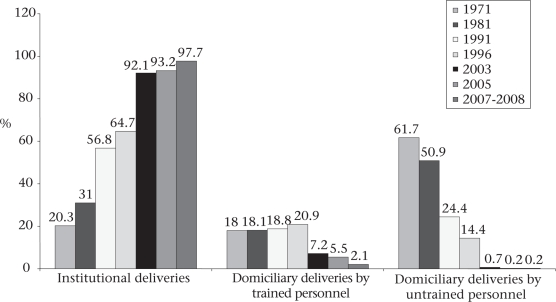
Increase in institutional deliveries from 1971 to 2007/2008

Promotion of institutional deliveries was a conscious effort of the Government of Tamil Nadu, along with improvement in the quality of care given to pregnant women during the antenatal, natal and postnatal periods. The NFHS 3 conducted in 2005 showed that the rate of institutional deliveries was 90.7% whereas more recent data on management information system (MIS) of the Government showed that the rate has increased to 97.7% in 2007-2008. Tamil Nadu has witnessed consistently increased rate of institutional deliveries from 20% in 1971 to 97.7% in 2007. Figure 1 shows the trends in institutional deliveries. Institutional deliveries have increased most likely due to various efforts by the Government of Tamil Nadu. As per the national norms, the Government deployed qualified Auxiliary Nurse Midwives (ANMs)/Village Health Nurses (VHNs) at the rate of one for 5,000 rural people and one for 3,000 people in tribal areas for antenatal care (ANC) visits and other reproductive health needs; Tamil Nadu is one of the states in India, which follows this national norm strictly. Along with the deployment of community-level staff, the Government also strengthened the infrastructure of the Subcentres (SCs) for conducting antenatal care clinics, deliveries, immunization services, treatments for minor ailments, and for provision of spacing methods of contraception. For further strengthening of the technical skills of VHNs, the Directorate of Public Health and Preventive Medicine periodically conducted various skills-building training programmes.

Source of data: Presentation of Dr Padmanabhan made at Safe Motherhood Case Studies Stake-holder Meeting at Indian Institute of Management, Ahmedabad, in February 2008

The Government also strengthened Primary Health Centres (PHCs) and Community Health Centres (CHCs): 229 of 1,532 PHCs were upgraded as 30-bedded PHCs, with five medical officers, three staff nurses, two ANMs, an ECG machine, laboratory equipment, and other basic services. Staff nurses in these facilities were trained to conduct normal deliveries independently and to refer women to a higher level without much delay by giving some obstetric first-aid in the case of complications.

A large proportion of the PHCs in Tamil Nadu have two medical officers posted in the facility, one of which is a female doctor. Posting of female doctors at the periphery is assumed to have played a major role in the increased use of health services by rural women.

#### Antenatal care

ANC may help identify some problems of pregnancy early, which will help the ANM to refer the mother in advance to a higher institution for timely intervention (10). For the promotion of ANC and institutional deliveries, the Government introduced certain incentive packages since 1996. The VHN/ANM receives an incentive payment of Rs 50 per case if she provides at least five ANC check-ups and conducts institutional delivery. If only ANC is provided by the ANM but she refers the case for institutional delivery, she gets an incentive of Rs 25. It is anticipated that this health reform impacted on the improved quality of ANC and institutional deliveries. Table 4 shows various maternal health indicators for the state.

**Table 4.** Maternal health indicators for Tamil Nadu (%), 1992-2005/2006 (6,8,11)

Health indicator	NFHS 1 (1992-1993)	NFHS 2 (1998-1999)	NFHS 3 (2005-2006)
Any ANC	94.2	98.4	98.6
3 ANC visits	18.2	91.4	95.9
ANC given by doctor	70.6	84.3	83.6
ANC given by ANM/nurse-midwife/LHV	7.5	9.8	14.3
ANC given by health worker in the home	16.1	4.4	3.3
Others/*dai*/none	5.8	1.3	1.1
Postnatal check-up within 2 months of birth	-	53.0	91.3
Institutional delivery	64	79	87.8
Caesarean-section rate	7.64	15.8	25.0

ANC=Antenatal care; ANM=Auxiliary Nurse Midwife; LHV=Lady Health Visitor; NFHS=National Family Health Survey

Receipt of three antenatal check-ups is nearly universal (96%). ANC received from the ANMs was low but is increasing, suggesting the need for the strengthening of midwifery services in the state.

#### Delivery care

The number of institutional deliveries is rapidly increasing in the state (discussed below) as is delivery by caesarean section.

#### Postnatal care

Recognizing the necessity of postnatal care (PNC), the Government of Tamil Nadu has mandated at least three PNC visits for each mother. Data of the NFHS 3 showed that PNC given by Tamil Nadu within two days of birth, is 87.2%, and 91.3% receive PNC within 42 days. Tamil Nadu is one of the topmost states other than Kerala and Goa with regard to care given during the postnatal period.

### Various innovations in Tamil Nadu for reduction of maternal mortality

The state has recognized that the following three interventions are needed to reduce maternal mortality: (a) surveillance and audits of maternal deaths, (b) continuum of care from the community to the first referral-level health facility to shorten the three delays, and (c) round-the-clock quality EmOC facility at the First Referral Units (FRUs).

#### Surveillance of maternal deaths and estimating the actual number of maternal deaths

**Fig. 2.**
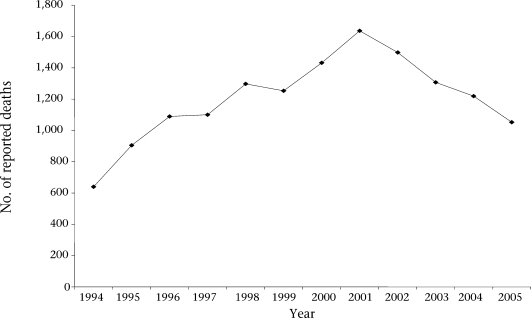
Maternal deaths reported during 1994-2005 (12)

To identify the reasons behind maternal deaths, the state started compulsory audit of all maternal deaths occurring in the state since 1994. The system became fully established when the Government of Tamil Nadu issued an order in 2004, stating that all maternal deaths should be audited. It mandates that each maternal death be reported to the MCH Commissioner within 24 hours of occurrence, irrespective of place of death—public facility or private nursing home or during the time of transit. Maternal deaths are reported by ANMs, medical officer posted at the periphery, FRU or non-government hospitals, district public-health nurse, and Deputy Director of Health Services. Investigations of maternal deaths are carried out through community-based maternal review (verbal autopsy) and facility-based maternal death reviews/clinical audits as described below. Figure 2 shows the increase in reporting of maternal deaths from 1994 to 2005.

Over the years, the number of reported maternal deaths increased from 640 in 1995 to 1,600 in 2001, followed by a decline in deaths registered.

#### Community-based maternal reviews (verbal autopsy)

Information on maternal deaths is obtained through telegram/fax/email, and medical officers perform the follow-up investigation within 15 days. This investigation tries to document specific circumstances that led to maternal death, including the first and the second delay in the community. Members of health staff are re-assured that the review of death is not a fault-finding exercise to punish individuals but to improve the system of care.

#### Facility-based review of maternal deaths

The second part of the review of maternal death is carried out in the facility where the woman was treated and/or had died. In the facility-based review, the causes, treatment given, and circumstances of deaths are investigated to see whether these deaths could have been avoided. This addresses the third delay and quality of care in a facility.

The findings of these reviews are placed before the Medical Death Audit Committee on a monthly basis. Minutes of the committee meetings are placed before the District Reproductive and Child Health (RCH) Committee chaired by the District Collector, who also receives relatives of the deceased who give their account of the events. Minutes of the review meetings are sent to the MCH Commissioner.

Positive outcomes of the maternal death verbal autopsy system are greater accountability of service providers, advanced information to referral centres, better coordination between referring and referral institutions, and a very few unrecorded referrals. Reviews of maternal deaths have indicated several problems in the healthcare-delivery system which led to maternal mortality. Examples include mal-distribution of FRUs in the state, shortage of staff at the FRUs, and unnecessary multiple referrals.

#### Near-miss case audit

‘Near-miss' events are defined either as acute obstetric complications that immediately threatens the survival of a woman but do not result in her death by chance or because of the hospital care she received during pregnancy, labour, or within six weeks after the termination of pregnancy or delivery. Besides maternal death audits, near-miss audits provide additional valuable information on whether the provider was able to handle the complication properly or not. In the effort to continually improve health services for mothers, the Government of Tamil Nadu started training medical officers to carry out near-miss audits, begining from May 2008.

Based on the reviews of maternal deaths and other evidence of how to reduce the MMR, the Government of Tamil Nadu has initiated various programmatic improvements as summarized below.

#### Enhancement of skilled care providers in rural areas

Generally, medical officers of PHCs do not stay at the PHC village and, hence, are not available for conducting deliveries during the night. To overcome this problem and to promote safe deliveries at the institutional level on a 24-hour basis, the Government of Tamil Nadu, during RCH I programme (1997-2004), piloted a scheme of appointing and contracting with three staff nurses (3½ years trained with diploma in general nursing and midwifery) to provide 24-hour delivery services in 90 PHCs in remote rural areas. These staff nurses receive a consolidated pay of Rs 2,500 (US$ 59.52) every month plus a payment of Rs 25 (US$ 0.59) as an incentive for each delivery conducted. They assist normal deliveries, take care of sick newborns, and do emergency referral to the FRU when an emergency arises. They also train VHNs/ANMs (VHNs—1½-year trained) to conduct deliveries at the SC and assist medical officers for minor operations. These staff nurses will be absorbed into regular employment in the health department after 2-3 years of service in the PHC.

Analysis of performance at one of the pilot intervention PHCs at Madurai revealed that the staff nurses conducted around 15.5 deliveries per month compared to 4.2 deliveries per month in the PHCs where the staff nurse model was not piloted [Source: Presentation of Dr Padmanaban made at Safe Motherhood Case Studies Stakeholder Meeting at Indian Institute of Management, Ahmedabad, in February 2008]. Table 5 shows the delivery performance of a PHC in Theni, another model intervention district. The model of posting contractual staff nurses at the PHC has improved the delivery performance in the PHC of Theni district by three-fold between 1999 and 2007.

**Table 5.** Annual percentage distribution of deliveries in Theni district, Tamil Nadu, 1999-2005

Place of delivery	1999-2000	2000-2001	2001-2002	2002-2003	2003-2004	2004-2005
Subcentre	9.8	9.7	10.8	8.7	11.1	11.4
Primary Health Centre	4.4	5.3	8.3	10.6	13.7	14.4
General hospital	18.7	19.72	21.2	25.9	27.97	30.88
Private nursing home	42.0	41.7	42.2	39.2	38.5	38.6
Domiciliary service sources	25.17	23.58	17.48	15.52	8.71	4.76

Source of data: Presentation of Dr Padmanabhan made at Safe Motherhood Case Studies Stakeholder Meeting at Indian Institute of Management, Ahmedabad, in February 2008

Table 5 also shows that the percentage of institutional deliveries started increasing in 2001-2002, especially in the PHCs. As the results of posting nurses to the PHCs in pilot districts were promising, the Government of Tamil Nadu decided to scale up this intervention in all the PHCs throughout the state during the RCH 2 programme. To do this, the Government hired additional 2,200 nurses on a contractual basis to staff selected PHCs. To support the staff nurses, two cleaning staff members are posted on the basis of a consolidated salary, and one driver is available round-the-clock in the facility to transport women with labour-pain cases to a referral site in the case of a complication during delivery at the PHC. In April 2008, the Government extended the ‘three staff nurses model' of 24-hour PHCs to all the 1,532 PHCs.

Table 6 shows the results of this intervention for Tamil Nadu. In 2007-2008, around 17.9% of deliveries (n=1.97 lakh of total 11 lakh deliveries in the state) took place at the PHC level; the number of domiciliary deliveries and those in private nursing homes has declined. During December 2008, the percentage of deliveries conducted in the PHCs increased to 25% of the total deliveries. There is a shift of deliveries from the private sector to the Government PHCs.

**Table 6.** Annual percentage distribution of deliveries in various facilities, Tamil Nadu, 1999-2007/2008 (12)

Place of delivery	1999-2000	2000-2001	2001-2002	2002-2003	2003-2004	2004-2005	2005-2006	2006-2007	2007-2008∗
Health Subcentre	5.2	5.3	5.7	5.9	6.7	7	6.9	5.9	5.3
Primary Health Centre	4.8	7.9	6.6	6.3	7	7.3	7.6	7.7	17.9
General hospital	36.6	34.5	37	38	38.5	39.6	40.6	41.8	40.8
Private nursing home	37.2	39.9	38.6	39.9	40.1	40.3	40.5	41.2	35.7
Dom. Total	16.1	14.4	12.1	9.9	7.7	5.7	4.4	3.4	1.0

∗Number of deliveries till February 2008; Dom=Domiciliary

#### Attracting doctors and staff nurses to work in rural areas

Common experience in many parts of India, including Tamil Nadu, is that the emergency services owned and run by the Government, such as ambulance services, are inefficient and not user-friendly, largely because of bureaucratic procedures of management and unionized unaccountable drivers and workers. Most state governments feel that trying to improve such government systems are very difficult. Hence, outsourcing was considered as an attractive alternative. The Government of Tamil Nadu pilot-tested this outsourcing alternative by contracting a non-governmental organization (NGO) (Sivanilyam Society) to run ambulances through the funds from the RCH project in Theni district. As part of the contract, the Government provided two ambulances, communication and life-saving equipment free of charge. The NGO provided salary of driver, maintenance of vehicles, fuel charges, and insurance. The NGO was allowed to recover the expenditure by charging Rs 5 per km from all patients. It was expected to offer services free of charge to 10% of cases (supposedly the poor), and all accident emergencies were transported free of charge.

A committee comprising officials from the State Government and NGO monitors the scheme. In Theni district, around 30-45 emergency cases every month are transported, of which 30% are obstetric emergencies (13). The ambulance is expected to provide quality service with timely life-saving first-aid services. The public was made aware of this intervention through various information campaigns. With the success of this project in Theni district, the State Government decided to expand the service throughout Tamil Nadu.

Under the Tamil Nadu Health Systems project (TNHSP), funded by the World Bank in 2005, the State Government developed a public-private partnership (PPP) with several NGOs to provide emergency transportation service in many districts. Under this partnership, the TNHSP provided equipped ambulance and running costs to the selected NGOs. Other minor expenses incurred for running the ambulance are borne by the NGOs. The NGOs met these expenses by charging nominal user-fees from those above the poverty-line. A monitoring and evaluation committee has been set up to evaluate the performance of these NGOs. A central control-room has been established in each district in the state with a common toll-free number of 1056. Based on the experience of Andhra Pradesh, the Government of Tamil Nadu, like the Government of Gujarat, is now switched over to the Emergency Management Research Initiative (EMRI) as PPP initiative to provide state-wide sophisticated effective ambulance service and established central call centre with toll-free number 108.

#### Increasing availability of specialists at FRUs

Despite the fact that most deliveries in Tamil Nadu are institutional, the MMR was around 140 per 100,000 livebirths in 2004. Policy-makers were very concerned about this issue and felt that ensuring institutional delivery alone would not reduce maternal mortality further. International evidence showed that adequate referral facilities with EmOC are required for dealing with complications effectively (15). One critical bottleneck in ensuring EmOC in rural areas was lack of specialists (obstetricians/gynaecologists, and anaesthetists). Hence, it was observed that many deaths take place among referrals from rural areas to city hospitals. The unavailability of blood in the FRUs was another important reason for maternal deaths.

To address the problem of non-availability of specialists, the State Government empowered the FRUs, starting in 2001, to contract local anaesthetists who are in private practice or retired. Initially, this was permitted at the FRUs for providing EmOC, including caesarean sections, hysterectomies, and other gynaecological surgical cases (15). Private anaesthetists were paid Rs 1,000 for each caesarean section and other emergency obstetric operations, including Rs 100 for conveyance. Later, anaesthetists could also be hired at the PHC for conducting tubectomies, where operating theatres (OTs) are functional on specific days (16). The contract is not a written formal contract document. However, the available private anaesthetists are listed and called as and when needed. Table 7 shows that an increasing proportion of FRUs performed more than 10 caesarean deliveries per month. Thus, the hiring of private anaesthetists has increased the access to EmOC, especially caesarean sections at the FRUs.

**Table 7.** Percentage of FRUs (n=163) providing caesarean sections by number of caesarean sections per month, Tamil Nadu, 1998/1999-2003/2004

No. of caesarian sections conducted per month	1998-1999	2000-2001	2001-2002	2002-2003	2003-2004
Nil	40.5	46.6	35.6	29.4	27.6
<5	30.1	24.5	33.7	29.4	26.4
5-10	10.4	8	8	17.3	14.7
>10	19	20.9	22.7	23.9	31.3

Source of data: Padmanbhan P, Desikachari. Personal communication. 2007

#### Establishment of comprehensive emergency obstetric and newborn care centres with round-the-clock emergency obstetric and newborn services

As it was realized that it was not possible to make all the 163 FRUs throughout the state fully functional on a 24-hour x 7-day basis given the limited staff, the Government of Tamil Nadu under the RCH 2 programme identified two hospitals in each district to provide round-the-clock comprehensive emergency obstetric and newborn care (EmONC) services. These hospitals were chosen in such a way that, from any lower-level facility, less than two hours is required to reach the site. These comprehensive EmONC centres were equipped to handle all kinds of obstetric and neonatal emergencies, including a dedicated operation theatre to handle obstetric emergencies, blood-bank, and laboratory services available round-the-clock. Equipment, manpower, and infrastructure were provided by the TNHSP funded by the World Bank, and operationalization and certification were done under the RCH project. Fully-functional centres are certified and accredited by the Government so that the managers and service providers of these centres are accountable for providing round-the-clock emergency services. By 2009, 125 hospitals have been accredited as comprehensive EmONC centres. As per the state norm, each such centre will have four obstetricians/gynaecologists, two general surgeons, four paediatricians, and two anaesthetists in specialist cadre and staff nurses who are trained in OT, blood-bank, and basic EmOC services.

#### Special programme for conditional cash transfer to pregnant women

Tamil Nadu has been fortunate in that there has been sustained political will to bring about favourable reforms not only in the health field but also in the social and economic spheres. Since 1989, the conditional cash-transfer scheme of the State Government—called Dr. Muthulakshmi Reddy Memorial Maternity Assistance Scheme—provides poor pregnant women with cash assistance of Rs 1,000 (US$ 23) per month for six months, which includes three months prior to delivery and after delivery. The assistance is meant for compensation for loss of wages. The purpose of giving the money is to provide for additional nutrition to the mother to prevent anaemia and low-birthweight babies. The total cost of this scheme is Rs 300 crore (US$ 75 million) per year. For many welfare programmes, the State Government identifies families below the poverty-line using specific criteria. Women of these families get the above benefit. This scheme has been playing a vital role in promoting woman and child welfare. Under this scheme, 441,095 pregnant women have so far benefited. No systematic evaluation of the scheme has been made to measure health benefits.

#### Increasing availability of blood

In some district hospitals and FRUs, blood-bank is available but blood-bank technicians are not in adequate numbers. Hence, the Government trained all doctors, staff nurses, and laboratory technicians working in comprehensive EmONC centres on blood-grouping, cross-matching, transfusion of blood, and management of transfusion reactions. In all the comprehensive EmONC centres, the plan is to establish blood-banks or blood-storage units to function round-the-clock. All blood-banks/blood-storage centres will be networked for information exchange and transfer out/in of required blood at any given point of time.

The list of voluntary donors/donor organizations, along with their telephone numbers, will be available in the blood-banks/blood-storage centres in addition to help-line centres (control room for ambulance services) in the office of the Deputy Director of Health Services.

#### Birth-companion programme to improve social support during delivery

Traditionally, women delivered in the home are surrounded by their family members who provide moral support during the delivery. Continuous support of a female relative to a woman during childbirth in an institution can also give a woman moral support and has proven beneficial to labour outcomes as per Cochrane review (17).

In India, it is a common hospital rule that the relatives of the woman accompany her up to the labour-room but are then not allowed to enter. As the number of staff members is few and the number of births is high, no one is available to provide such moral support to a woman in labour. Although if there is a staff nurse in the labour-room, she remains busy with her clinical or other administrative work. Besides, she may lack skills and motivation to give moral support or may work in a short-staffed environment. Many women prefer delivery in the home due to lack of social support in labour-rooms of hospital.

Christian Medical College, Vellore Hospital—a pioneering NGO medical institution in Tamil Nadu—started a birth-companion programme in 2002. This model was accepted in the Chennai Municipal Corporation in two 24-hour x 7-day EmOC facilities, although there was initial resistance from doctors and staff nurses because they feared that relatives might intervene during the birthing process. A one-day sensitization programme was carried out at each hospital for the labour-ward staff nurse and doctors in an effort to overcome opposition to the scheme. During July 2004, a government order was issued to scale up this programme for all government hospitals in the state (18), and doctors and staff nurse were made aware of this initiative taken by the Government.

The following criteria were laid down for selection of the birth companion: (a) a female relative of the mother; (b) who had undergone the process of labour earlier, and; (c) who will stand with the mother during her entire labour and who should not interfere the services done by the staff nurse or the doctor.

Theoretical benefits of this scheme are shortened duration of labour, less medication, less use of intravenous fluids, lesser interventions, such as forceps deliveries and caesarean sections, and reduced informal payments in the hospital. Although the Government of Tamil Nadu did not measure the benefits of the birth-companion programme, the impression is that it helps women.

#### Maternity picnic

To reduce the apprehension of birth in hospital, on selected days, pregnant women are invited to the PHC/Hospital for ANC to see the facilities, including the labour-room and enjoy a free lunch. Social functions, such as Bangle Ceremony, are sometimes organized for pregnant women according to local traditions. This helps make the hospital a welcoming and familiar place for mothers.

#### Tamil Nadu Health Systems Project

World Bank has funded the TNHSP for a five-year period (2005-2010). The main objective of the project is to support the efforts of the Government for improving healthcare in the state. In the first phase, the project had interventions in the districts of Dharmapuri, Theni, Kanyakumari, Khrishagir, and Pudikottai. The project now covers all the 270 secondary hospitals in Tamil Nadu. The project has planned interventions in the following areas:

Reduction in maternal and neonatal mortality—Establishment of comprehensive EmONC centres for handling obstetric and neonatal emergencies: The TNHSP is supporting these comprehensive EmONC centres with additional buildings, equipment, and manpower. Equipment costing three million dollar are being provided to the comprehensive EmONC centres. The total number of comprehensive EmONC centres has increased from 62 in 2004 to 125 in 2009.

The other major activities include strengthening of hospitals, tribal health programmes, biomedical wastes management, etc.

#### Urban health programme

In addition to the rural health programme, the state has also given importance to the urban health programme to look after the health of 27 million urban people settled in the six metropolitan cities. There are various urban health centres in the state, including the Postpartum Units, which are usually attached to the district hospitals or CHCs. There are 65 urban health centres to take care of the needs of urban people. The Chennai Municipal Corporation runs its own urban welfare clinic, which functions like a subdistrict hospital. The facility has 81 beds, a fully-functional neonatal care unit, an obstetric OT, Prevention of Parent to Child Transmission (PPTCT) Centre, and Voluntary Counselling and Testing Centre (VCTC).

#### Teamil Nadu Medical Services Corporation Limited

To strengthen the logistics management system of healthcare, the Government of Tamil Nadu has established a Tamil Nadu Medical Services Corporation (TNMSC) which became functional in January 1995. It is structured as a government-owned company and registered under the Companies Act. It serves as an apex body for the purchase, storage, and distribution of medicines, other supplies, and surgical instruments for various government medical institutions in the state. It also renders other services, such as supplying equipment to hospitals and maintaining its own CT scan centres on the premises of some selected government hospitals. In addition to procurement and maintenance, the TNMSC also maintains the website of the Ministry of Health and Family Welfare where the public can interact with doctors and other medical staff through health-chats. Managing Director looks after the day-to-day administration of the corporation. Efficient professionals from faculties of medicine, pharmacology, etc., employed on deputation, work in the corporation to assist the Managing Director in technical matters. The corporation ensures that all facilities have regular supply of required medicines and consumables. No state other than Tamil Nadu has such an efficient and transparent medicine supply.

#### Family-planning services in Tamil Nadu

Family planning has been a high-priority programme in Tamil Nadu for many years. The couple-protection rate in Tamil Nadu has increased rapidly in the recent past (Table 8). The presence of female doctors has helped rural women in Tamil Nadu to have access not only to family-planning services but also to general healthcare, including maternal healthcare.

**Table 8.** Percentage of couples using various family-planning methods, Tamil Nadu (NFHS 1, 2, and 3) (6,8,11)

Family-planning method	NFHS 1 (1992-1993)	NFHS 2 (1998-1999)	NFHS 3 (2005-2006)
Female sterilization	33.3	45.2	55.0
Male sterilization	1.4	0.8	0.4
Intrauterine device	6.1	2.5	2.1
Condom	3.0	1.5	2.3
Pill	0.9	0.3	0.2
Total modern methods	44.7	50.3	60.0

Increasing the couple-protection rate may also have contributed to reduction in maternal deaths. Female sterilization has increased over the period compared to male sterilizations. However, a low use of temporary methods and male sterilization remains a matter of concern.

### Increased access to safe abortion services

In Tamil Nadu, most medical termination of pregnancies (MTPs) are carried out by surgical techniques requiring trained personnel. Only medical doctors who had undergone training and certification are permitted to do MTPs. Mid-level care providers, such as nurses and ANMs, are not allowed to be trained for providing MTP. The manual vacuum aspiration (MVA) by doctors was piloted in five districts and seven medical colleges, besides the Chennai Corporation under the RCH 1 programme in 1997-2002. This pilot scheme provided 6,000 MVA services in 2007, and under the new programme of the National Rural Health Mission (NRHM) (19), all 385 block PHCs will provide MVA services. The MVA technique, if implemented properly, can reduce the number of unwanted pregnancies, which, in turn, can help reduce maternal mortality and morbidities to a significant extent.

In India as a whole, 1.3% and 4.5% of all pregnancies reportedly resulted in induced and spontaneous abortions respectively. In Tamil Nadu, on the other hand, the comparable figures were 4.3% and 7.0% (20,21). Table 9 gives the number of MTPs and MTPs with sterilization for 2004 to 2007.

**Table 9.** MTP performance in Tamil Nadu, 2004/2005-2007/2008

MTP with					
Year	Total MTPs	Sterilization	IUD	Oral pills	Only MTP
2004-2005	72,710	37,353	3,898	1,249	30,210
2005-2006	71,128	32,350	4,274	1,292	33,212
2006-2007	67,315	28,958	3,149	973	34,235
2007-2008 (P)	61,435	26,154	2,493	1,154	31,634

Source of data: Official health statistics of Government of Tamil Nadu; IUD=Intrauterine device; MIPS=Medical termination of pregnancies; P=Provincial

More than 40% of the total MTPs performed was followed by permanent sterilization. The insistence in government institutions on permanent sterilization, along with MTP, violates rights of women to reproductive choice and also drives women to unsafe abortions in the hands of quacks. There is an urgent need to de-link abortion services from provision of permanent methods of contraception. Although there has been a recent decline in numbers of MTPs (Table 9), the number of clinics approved for conducting abortions or MTPs has risen by more than four times in 13 years from 1980 to 1993. Table 10 shows the number of institutions approved by the Government to provide MTP services in both public and private sectors.

**Table 10.** Number of institutions approved for doing MTPs, Tamil Nadu, 1993

Total number of institutions approved for MTPs	1,583
Government institutions, including local bodies	541
Non-government institutions	1,042

Source of data: Official health statistics of Government of Tamil Nadu

Quite likely, many abortions go unrecorded in official statistics. Of the official number of abortions, the proportions in the government sector (government hospitals and urban health posts, and PHCs) varied from 55% to 60% with private nursing homes providing the remainder. It is likely that many unregistered doctors perform abortions in the state.

Results of analysis of maternal death audits showed that more than 6% of (around 84) deaths were due to abortion-related complications, indicating that unsafe abortions still take place in Tamil Nadu, perhaps, due to lack of access to safe abortion services. To address this issue, the following measures were proposed by the Government: (a) Increase the number of health facilities providing MTP services; (b) Encourage private and NGO sectors to establish quality MTP services; (c) Train an adequate number of medical officers for providing MTP using MVA method; and (d) Counsel mothers by the ANMs, *Anganwadi* workers, and link workers for selecting safe abortion services when needed.

### Organizational structure of the Ministry of Health and Family Welfare of Tamil Nadu

There is a lot of political commitment for social services, including healthcare in the state. Historically, social reformers and political leaders have given more importance to health. This has helped raise awareness levels, enlarged the demand for quality health services, and improve the use of available health infrastructure. The state issues official government orders for all activities carried out for improvement in maternal healthcare. The public are made aware through various advocacy campaigns. The commitment of the Government to provide health services from the periphery level is underscored by its having ensured almost full staffing at rural health services.

**Fig. 3.**
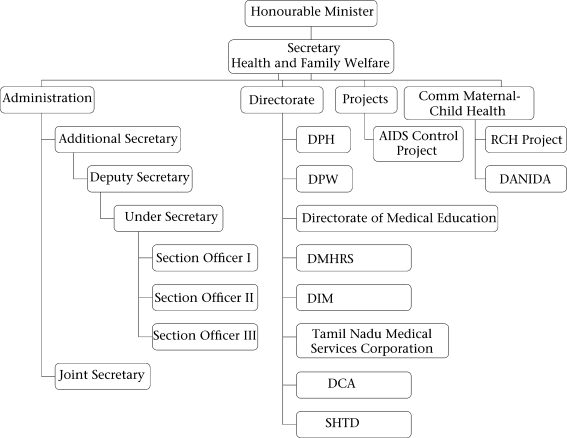
Organogram of Ministry of the Helth and Family Welfare

The Minister for Health and Family Welfare is the primary authority in taking decisions in matters relating to health services. The Secretary of Health supports him. Under the Health Secretary, there are eight directorates, the head of external aid projects, and health officers of corporations. Figure 3 provides the organogram of the Ministry. Dedicated directors head each directorate. This contrasts with Gujarat, for example, where one Commissioner of Health and Family Welfare under the Secretary of Health is in charge of six directorates, with six additional directors to assist him. The administrative structure in Tamil Nadu at the state level is much better-developed than in other states, which may be one of the reasons for better management of maternal health programmes.

Comm=Commissioner; DCA=Drug Control Authority; DIM=Directorate of Indian Medicine; DMHRS=Directorate of Medical and Rural Health Services; DPH=Directorate of Public Health and Preventive Medicine; DFW=Director of Family Welfare; RCH=Reproductive and child health; SHTD=State Health Transport Department

#### Directorate of Medical Education

The Directorate of Medical Education (DME) was separated in 1966 from the Directorate of Medical Services. The responsibility of the DME is to implement teaching, training and research programmes in the medical field and patient-care services in teaching hospitals. The Director regulates the functions of government and private self-financing medical/dental/paramedical colleges. At present, the state has 15 government medical colleges; seven private medical colleges, one government dental college, and seven private dental colleges are functioning under the DME. The Government has taken a policy decision to establish government medical college in each district, and 10 more medical colleges will be established in a phased manner.

#### Directorate of Medical and Rural Health Services

In 1956, the Family Welfare Programme was taken up by the Department of Medical Services to promote the health of the people, particularly mothers and children. The department, a big unit at the time of inception, was divided into various smaller units for effective functioning. In 1983, the Directorate of Family Welfare (family planning + maternal and child health) was separated from the Directorate of Medical Services and Family Welfare for better coordination and implementation of the family welfare programme. From 1999 onwards, this department—renamed as the Department of Medical and Rural Health Services—was entrusted with the responsibility of rendering medical care to the public through the non-teaching medical institutions (22).

The Director of Medical and Rural Health Services is in charge of planning and implementation of all programmes of medical services through the 29 district hospitals, 155 taluka headquarters hospitals, 80 non-taluka hospitals, 12 dispensaries, 11 mobile medical units, seven women and children hospitals, two tuberculosis (TB) hospitals, two TB clinics, and seven leprosy hospitals. The Director of Medical and Rural Health Services also looks after implementation of various acts, such as the Human Organ Transplantation Act, Private Clinical Establishment Regulation Act, Prenatal Diagnostic Techniques Act, Recognition of Private Hospitals, etc.

Either the Joint Director or Deputy Director of Medical and Rural Health Services and Family Welfare administers 29 of the 32 revenue districts in the state, except Chennai city. For administrative efficiency and better supervision, health services in these 29 districts have been divided into 42 district-level health units, headed by 42 Deputy Directors of public health—all postgraduate in public health. In many states in India, district-level public-health services are headed by officers who are medical doctors but not public-health specialists. Table 11 gives statistics of the existing health infrastructure in the state.

**Table 11.** Health infrastructure in Tamil Nadu, 2007 (23)

Facility	Total no. of facilities	Key staff sanctioned
Subcentres	8,706	1 ANM (village health nurse)
Primary Health Centres	1,421	Medical officers, staff nurses
Rural Family Welfare Centres	382	Medical officer/staff nurse
Non-taluka hospitals (CHCs)	79	Specialists, staff nurses
District hospitals	29	Specialists, including obstetrician
Taluka headquarters hospitals (First Referral Units)	162	Specialists and staff nurses
Medical colleges	11	Professors, tutors
Teaching hospitals	40	Super specialists, medical officers, staff nurses
Specialty hospitals	8	Super specialists
Postpartum centres	114	Medical officer/staff nurse
Urban Family Welfare Centres	104	Medical officer
Urban Health Centres	193	Medical officer/specialists
Voluntary organizations	27	Medical officer/specialists
Private approved nursing homes	1,645	Obstetrician and specialists

ANM=Auxilliary Nurse Midwife; CHCs=Community Health Centres

#### Directorate of Public Health and Preventive Medicine

The Directorate of Public Health and Preventive Medicine implements various national and state-level health programmes. It provides primary healthcare services through a network of 1,532 PHCs and 8,706 SCs spread over the entire state. The major activities of this department include maternity and child-health services, immunization of children, control of communicable diseases, etc. The main activities include training of multipurpose health supervisors, training for medical officers, including induction training, management training, foundation training, pre-service training, and orientation training to health inspector. Other than these trainings, the Directorate also runs public-health training for continuing education.

The Health SC is at the periphery providing outreach healthcare to the rural population. As per norm of the Government of India, the SCs are established and maintained at the rate of one SC for every 3,000 people in the hilly, tribal and difficult terrain areas and one for every 5,000 people in other areas. Under the DANIDA-assisted healthcare project in four districts, new SCs were constructed with all necessary facilities required for the smooth functioning of the health system. Seventy-five percent of the SCs are working from their own buildings. Around 60% of village health nurses (ANMs) stay at the headquarters. Both statistics are much higher than in other states in India. A village health nurse assisted by a trained *dai* operates an SC and provide preventive and primitive services to the population.

### Issues and challenges

#### MMR is still high

The state has undertaken various initiatives to reduce the MMR, and hence, it has come down to 90 per 100,000 livebirths, one of the lowest in India. However, compared to other countries, such as Sri Lanka, Malaysia, and China, the MMR could be further reduced. A recent newspaper report claimed an average of 10 deaths per month out of 4,000 deliveries in Madurai district (one of the project districts under the TNHSP) which translates to an MMR of 250 per 100,000 livebirths (24). If this is true, it indicates that there are still pockets of high maternal mortality in Tamil Nadu. The reasons for high maternal mortality could be many, including the lack of access to high-quality skilled care and EmOC, poor nutritional status, including high levels of anaemia, and improper referral in the case of emergency. Thus, there is a need to analyze the MMR district-wise and focus on those districts which have a high MMR.

#### Over-medicalization of childbirth

The NFHS data showed that the rate of caesarean sections has been rapidly increasing. Given the programme to hire private anaesthetists to help with cesarean sections in rural areas, it may be possible that unwarranted and unjustified caesarean sections have started happening. This may be more so in the private sector. Hence, along with ensuring availability of EmOC, it is necessary to review indications of caesarean sections and the rate of population-based caesarean-sections in different geographical areas. Along with such measures, the use of partograph could be introduced in the state to guard against over-medicalization of delivery care.

#### Finding doctors for rural areas

Currently, the state is filing up posts of medical officers in the periphery by attracting doctors who just finished medical college education. The main attraction is quotas set aside for postgraduate education through the government systems. However, the State Government officers feel that, with the increase in salaries in the private sector, the state may not be able to attract doctors to work in rural areas, and this will hamper EmOC services in future. A second major challenge is that, even when doctors are posted in rural PHCs, they do not stay there and commute from urban areas. Their availability at night is, thus, very limited. The state may, therefore, have to think of developing a model of maternal care based on nurse-midwife in rural areas. Currently, there is no qualified midwifery cadre in the state.

### Challenges in service-delivery infrastructure

Despite substantial improvements in maternal health services in Tamil Nadu, the major challenges remain in improving the quality of infrastructure and services in rural areas for maternal health. A one-day field visit to a few facilities in July 2006 by the research team provided valuable insights into some remaining challenges. These are summarized below.

#### Observation in Kancheepuram District Hospital (comprehensive EmONC facility)

The Government certified the facility as a comprehensive EmONC centre in 2005. The hospital has four obstetricians, four surgeons, four anaesthetists, four paediatricians, and 4-5 ambulances in working condition. After regular hours, the specialists are available at the hospital on an on-call basis. The maternity section is housed in a separate building on the ground floor, with a dedicated OT for obstetric and gynaecological surgeries. The hospital carries 500-550 deliveries per month, and the rate of caesarean sections of the hospital is around 30%. The facility has a blood-bank which is housed near the maternity section ensuring regular supply of blood in the case of emergency.

The key problems observed included:

• The facility is housed in an old building needing repair and renovation.
• The post-operative patients (both gynaecological and obstetric cases) are kept together in the wards which is not ideal. The labour-room is not clean, the curtains are torn, and there are stray-dogs in the facility. The labour-tables do not have adequate mackintosh and are stained with blood, indicating lack of proper cleaning. Sweepers and other support staff do not follow the aseptic precautions for cleaning the OT area. Unused equipment was lying in the facility occupying a lot of space.
• The anaesthetist, although posted for full-time service, works as on-call basis after office hours which may lead to some delays.
• The neonatal care unit is located outside the maternal care unit in a separate building. There are no places for nursing mothers to take care of the baby in the neonatal care unit.

#### Observations in upgraded PHC (basic EmONC facility), Walajabhad

The current PHC at Walajabhad was upgraded to a 30-bed facility at a cost of more than Rs 40 lakh to serve about 42,000 people. The PHC was upgraded to admit patients for family planning and other minor surgeries and also to give first-aid to accident victims in the area but the wards are highly underused. Located on the main road near the bus-stand at Walajabhad, the PHC provides outpatient treatment. It has two buildings: the main building consists of an outpatient ward, labour-room, laboratory, duty doctor's room, pharmacy-room, and store-room to keep medicines and other equipment. The new building has 30 beds where the team found only one patient admitted for diarrhoea. The labour-room in the old PHC is small and crowded; if the labour-room is shifted to the new building, it could provide better care. The facility has round-the-clock electricity and water supply. The cold-chain equipment in the facility was functional. The staff include three medical officers in shift-duty ensuring round-the-clock services, two staff nurses, three ANMs (one post was vacant), one laboratory technician, one pharmacist, and one male nurse. The centre has one jeep and one ambulance for providing transport facility. The centre handles only normal deliveries, and complicated cases are referred to the district hospital in Kanchipuram. For performing sterilization procedures, the specialists come from the district hospital, and the medical officers assist them. The centre does not provide MTP and other emergency services. It holds a camp for Pap-smear testing every first and third Tuesdays, and every Friday, it holds ANC clinic and hypertension clinic. It maintains a referral register but there is no back-referral. The nurses are on a contract basis; after completing two years, on the basis of the performance, they will be regularized. During April 2005–March 2006, the village health nurses (ANMs) conducted 75 deliveries in the PHC, and 21 deliveries were conducted in the home.

The following key problems were observed:

• Although the PHC has three medical officers and two staff nurses, none of them stay in the campus or in the PHC village; all of them commute from the neighbouring town.
• The PHC does provide basic EmOC services.
• The facility is functioning as a medical facility for common ailments, and EmOC cases are referred to the district hospital.
• The labour-room in the old building is used but is congested while the new and spacious labour-room built in the adjoining new building is not used for conducting deliveries. It is used for storing materials. The new labour-room has an attached toilet which is not clean.
• The facility has a jeep which is not used for transfer of patients. It does not have an ambulance.
• A telephone is available but there is no standard protocol for its use in emergency. It is used for general communication purpose.

#### Observations in Subcentre, Musaravakkam

The SC, located near the village, is easily accessible by road. The ANM stays in the SC building and conducts deliveries there. During the visit, we saw a lady in labour admitted to the SC. The SC has basic facilities and equipment. The registers are kept very well, and information, education, and communication materials are also available. She also maintains a chart for the maternal-child health (MCH) and family-welfare achievement. The workload of the facility is high, and she expressed that there is a need of a support staff to help her. As there are no sweepers available through the government system, she hires local people for cleaning and pays them out of her pocket. It was seen that, with the limitation in the facility, the ANM is doing a good job.

The following key problems were observed during the visit:

• The building was not well-maintained.
• There is no electricity or water supply at the facility. Electric supply was cut because the payments have not been made for the electricity bills. The ANM reported that, during the summer season, it is very difficult to stand in the labour-room without electricity.
• The labour-table was stained with dried blood.
• There is no telephone facility in the centre.
• The ANM has reported about the problems at the facility to the higher officials but no action has so far been taken.
• The ANM relies on the public transport system for submitting the monthly reports and other routine activities. Although the Government has provided a two-wheeler, there is no provision for fuel and maintenance allowance; so, it remains unused.

Our field visit indicated that, although Tamil Nadu has made efforts to improve maternal care, there still is room for further improvements in quality. Tamil Nadu has already been working in this direction, and some changes have taken place since our visit in 2006. Many facilities, mainly PHCs and SC, have been renovated.

## CONCLUSIONS

The state of Tamil Nadu provides better health services, specifically MCH services, compared to other states in India. Due to political commitment and proactive administration, the indicators of maternal health have improved over the years. One of the key factors is the continuity of key top-level officers looking after MCH for a long time. The state is not only implementing various national health programmes in maternal health but also adding its own innovations to improve the programmes. Efforts to improve maternal health include improvements in availability of human resource, availability of drugs and supplies, improved management capacity, better monitoring of health services, and analysis of maternal deaths. All of these has resulted in better quality of services, improved care coverage, and lower maternal mortality compared to other states in India.

Despite this progress, Tamil Nadu has a long way to go in improving maternal health. However, it can provide a good role-model and roadmap for various states in India and neighbouring countries that want to reduce maternal mortality. The Government of India can take lessons from the innovations in this state to increase progress in achieving Millennium Development Goal 5.

## ACKNOLEDGEMENTS

This study was supported by funds provided by the Department for International Development (DFID), UK. Its contents are solely the responsibility of the authors and do not necessarily represent the official views of DFID. The authors also acknowledge the support of the Government of Tamil Nadu and the officers of the Department of Health and Family Welfare for their assistance in terms of facilitating the visit to the health facilities and also providing them with relevant data and other information for completing this paper.
